# Therapeutic Application of Exosomes in Inflammatory Diseases

**DOI:** 10.3390/ijms22031144

**Published:** 2021-01-24

**Authors:** Ju Hun Suh, Hyeon Su Joo, Eun Be Hong, Hyeon Ji Lee, Jung Min Lee

**Affiliations:** School of Life Science, Handong Global University, Pohang 37554, Korea; 22032004@handong.edu (J.H.S.); mecanum0342@handong.edu (H.S.J.); ghdtka37@handong.edu (E.B.H.); hjilee@handong.edu (H.J.L.)

**Keywords:** inflammation, autoimmune disease, immunomodulation, exosome

## Abstract

Immunomodulation is on the cusp of being an important therapy for treating many diseases, due to the significant role of the immune system in defending the human body. Although the immune system is an essential defense system, overactivity can result in diverse sicknesses such as inflammation and autoimmune disease. Exosomes are emerging as a state-of-the-art therapeutic strategy for treating an overactive immune system. Thus, in this review, we will thoroughly review therapeutic applications of exosomes in various inflammatory and autoimmune diseases. Finally, issues for an outlook to the future of exosomal therapy will be introduced.

## 1. Introduction

Immunomodulation is on the cusp of being an important therapy for treating many diseases, due to the significant role of the immune system in defending the human body. Although the immune system is an essential defense system, overactivity can result in diverse sicknesses [1].

Representative consequences of an over-functioning immune system are inflammation and autoimmune disease. Inflammation results from an immune response against harmful stimuli. Appropriate inflammation is needed to protect the body, but severe inflammation can cause dangerous, unwanted effects and impair tissue function at the disease site [2]. Autoimmune diseases develop when effector immune responses against the body’s own tissues are not properly controlled [3].

Exosomes are emerging as a state-of-the-art therapeutic strategy for treating an overactive immune system. Most cells secrete exosomes, which can have a potent immunomodulatory activity, depending on the type of cell from which they originate. Their activity can be controlled by cell culture conditions [4,5]. Because exosomes are a cell-free therapy, they have lower toxicity and generate less of an immune response than do cell-based therapies [6]. Additionally, exosomes have the potential to be a drug delivery system, because they protect internal biomolecules from degradation [7]. Thus, exosomes have drawn great interest for their potential clinical applications in treating diseases associated with abnormal immune regulation. 

Hence, in this review, we will discuss trends in exosome research related to inflammatory and autoimmune diseases. Possible therapeutic applications of exosomes will be summarized. Finally, an outlook to the future of exosomal therapy will be introduced.

## 2. Inflammation and Autoimmune Diseases

### 2.1. Inflammation

Inflammation is an immune response that defends the host against pathogens or damage. However, unregulated inflammation can cause many diseases. Inflammation is primarily mediated by innate immune factors, including cytokines, chemokines, and innate immune cells [8]. 

Inflammation can be classified into several types, including acute inflammation, chronic inflammation, neuroinflammation, and systemic inflammation. In many cases, inflammation is classified as acute or chronic depending on its strength or duration. Acute inflammation is the initial response to the stimuli. If such inflammation is not resolved or treated effectively, low-grade, long-term chronic inflammation occurs, continuing after the acute inflammation [9].

In general, inflammation is progressive [2]. Each stage advances to the next through the action of cytokines, signaling molecules that participate in immune cell interactions. The initial stage of inflammation occurs when immune cells recognize a pathogen or a form of damage through their pattern recognition receptors (PRRs). PRRs, which include Toll-like receptors (TLRs) and NOD-like receptors (NLRs), bind pathogen-associated molecular patterns (PAMPs), such as bacterial lipopolysaccharides (LPSs), or host-derived damage-associated molecular patterns (DAMPs), such as molecules released from a dying cell. Recognition of these ligands leads to the activation of inflammation-related transcription factors such as nuclear factor kappa-light-chain enhancer of activated B cells (NF-κB), activator protein 1 (AP1), cAMP response element-binding protein (CREB), CCAAT-enhancer-binding protein (c/EBP), and interferon-regulatory factor (IRF) [8].

As is well known, NF-κB is a key transcription factor in the inflammation signaling pathway [10]. NF-κB is retained in an inactivated form in the cytoplasm by binding with IκB. After PRRs interact with a PAMP or DAMP, NF-κB becomes dissociated from IκB. In turn, NF-κB moves into the nucleus, and promotes the expression of inflammation-related genes. Most of these genes encode pro-inflammatory cytokines or chemokines, such as interleukin 1 beta (IL-1β), IL-6, tumor necrosis factor-α (TNF-α), monocyte chemoattractant protein-1 (MCP-1), C-X-C motif chemokine ligand 1 (CXCL1), and CXCL10 [10]. Furthermore, NF-κB signaling can regulate the survival, activation, and differentiation of inflammatory T cells. Thus, NF-κB has a crucial role in inflammation.

**Figure 1:**
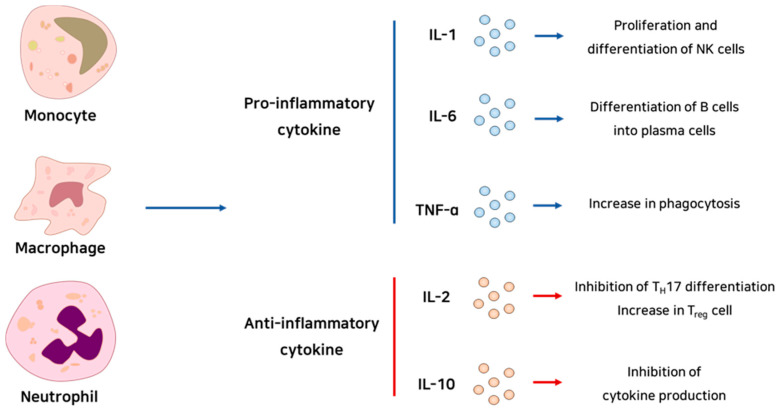
Schematic summary of immunoregulatory cells and cytokines.

Cytokines are the mediators of inflammation. They commonly have a role in signaling pathways related to immune cell proliferation, differentiation, and activation (Figure 1). Chemokines are chemotaxis-related signaling molecules that are co-expressed with cytokines. These molecules, produced by the leukocytes that gathered earlier at the site of infection or damage, can recruit inflammatory effector cells into the site by inducing integrin expression [11]. 

Inflammatory effector cells include mast cells [12], platelets [13], monocytes [14], macrophages [15], neutrophils [16], and inflammatory T cells [17]. These cells are recruited by chemokines; their proliferation, activation, and survival are controlled by cytokines. Furthermore, these cells not only participate in boosting inflammation, but also in repairing or regenerating tissue that has been damaged by inflammation.

Inflammation has gained attention because it can be caused by various factors other than infection. For example, a correlation between obesity and inflammation has been studied in great deal, in accordance with the increasing frequency of obesity [18]. Some studies show that obesity is accompanied by chronic low-level inflammation. In an obese state, inflammatory processes are activated in early stages of adipose expansion. Moreover, M1 macrophages, which are pro-inflammatory, are recruited into adipose tissue. Additionally, Kupffer cells (resident macrophages of the liver) and recruited macrophages exhibit increased production of inflammatory cytokines and chemokines, which increases insulin resistance in hepatocytes. Furthermore, obesity causes intestinal permeability to increase, which in turn causes the circulating LPS level to increase. This phenomenon is also related to type 2 diabetes [18]. Interestingly, anti-inflammatory agents are being used to treat type 2 diabetes [19]. 

Aging, as well as obesity, might also cause inflammation. In fact, the word ‘inflammaging’ has been suggested [20], indicating a status in which there are increased levels of inflammatory markers in the blood. Mechanisms through which aging could result in inflammation include increased gut permeability, alteration of the microbiota composition, increased cellular senescence, NLRP3 inflammasome activation, and increased oxidative stress. Such aging-induced inflammation can increase the likelihood of cancer, type 2 diabetes, dementia, sarcopenia, depression, and other diseases [20].

As mentioned above, intestinal permeability also has an important role in the immune response. Many researchers have found that the gut microbiota has an important role in the maintenance of intestinal permeability [21]. The representative chronic inflammatory disorder of the gut is inflammatory bowel disease, which includes Crohn’s disease and ulcerative colitis; this condition results from an abnormal immune response to commensal bacteria [22]. Thus, in gut inflammation, the importance of commensal bacteria is gaining attention.

### 2.2. Autoimmune Diseases 

The fundamental mechanism of autoimmune diseases is the imperfect elimination of self-reactive lymphocytes. Commonly, autoimmune diseases develop because of a combination of genetic and environmental factors. In some cases, infection and commensal microbes can influence disease occurrence.

Regulatory T cells (Tregs) play a key role in modulating the immune response [3]. Although the mechanism of the regulatory function of regulatory T cells is unclear, it has been known that cytokines such as IL-10, transforming growth factor beta (TGF-β), and IL-35 function as the means of regulatory function of the cells. Thus, if the number or function of Tregs decreases, defects in peripheral tolerance, allergic responses, and subsequent autoimmune diseases can develop. For example, in the model of autoimmune diseases, the number of T_H_1-like regulatory T cells increases. The increased T_H_1-like regulatory T cells possess reduced immunomodulation function. Thus, this indicates that the maintenance of the function of regulatory T cells is important in autoimmune diseases [3]. Several studies have indicated that type 1 diabetes, multiple sclerosis (MS), myasthenia gravis, rheumatoid arthritis, and other autoimmune diseases are caused by Treg deficiencies.

However, abnormal Treg function is not the only cause of autoimmunity. The autoimmune disease systemic lupus erythematosus (SLE) occurs because of the incomplete negative selection of immature B lymphocytes in the bone marrow. Imperfect B cell receptor editing in B lymphocytes can also cause SLE. Such B lymphocytes, which exhibit self-reactivity, produce autoantibodies [23]. In contrast to type 1 diabetes, which is an autoimmune disease in which the immune response is targeted against a specific organ (islet cells in the pancreas), SLE is generated by the loss of tolerance to systemically expressed self-antigens [24].

Host–gut microbiota interactions play an important role in the development of self-tolerance [22]. Interference with host–gut microbiota interactions increases susceptibility to autoimmune disease. Again, this effect occurs because of the role of the gut microbiota as a trainer of the host’s immune response.

**Figure 2:**
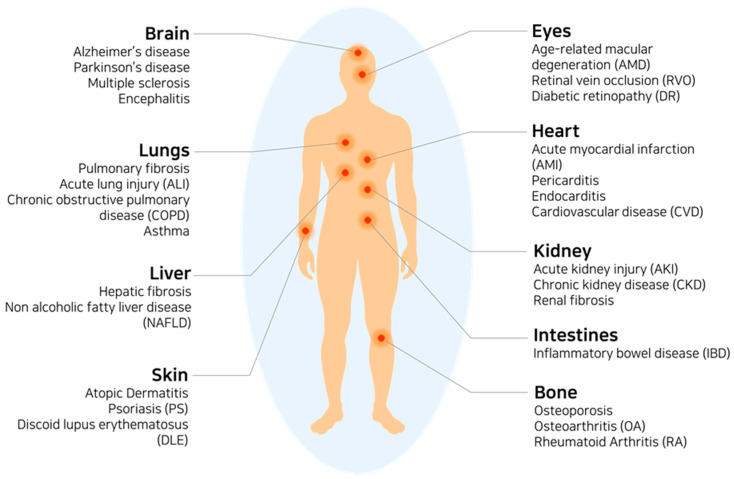
A schematic of the human body with inflammatory diseases.

Overall, inflammatory responses can lead to tissue damage in the inflamed area and, more importantly, dysfunction in organs. Inflammatory diseases are summarized in Figure 2, which can also be seen as therapeutic targets.

### 2.3. Treatment of Inflammation and Autoimmune Diseases

Inflammation and autoimmune diseases share a common pathogenesis. Both occur because of a failure to control the immune response of the host. Thus, treating both conditions involves targeting immune regulation.

To treat inflammation, anti-inflammatory agents that can modulate inflammatory signaling are usually used. Up-to-date, non-steroidal anti-inflammatory drugs (NSAIDs), which inhibit prostaglandin production, are widely prescribed to patients [25]. Additionally, studies on anti-inflammatory small molecules [26], serine protease inhibitors [27], microRNAs (miRNAs) [28], and natural products [29] are in progress. 

Therapeutics that block self-reactivity are being tested for autoimmune diseases. For example, monoclonal antibodies that recognize B cells are being exploited to treat autoimmune diseases [30]. These antibodies decrease the number of B cells and can alleviate disease severity. Administration of Tregs is also being tested [31]. In addition to these strategies, signaling pathway inhibitors [32], stem cell transplantation [33], and other approaches are being studied extensively.

However, for a drug to be effective, various factors must be considered. One of them is drug toxicity. NSAIDs have gastrointestinal toxicity [25], and small molecules and miRNAs have toxicity issues [26,28]. In some cases, therapeutic antibodies are too large to penetrate the desired tissue easily. Moreover, the antibodies target extracellular antigens only, limiting their use [34]. Mesenchymal stem cell treatment in inflammation and autoimmune diseases also faces challenges such as low cell survival rate, impaired paracrine effects, and poor homing capacity [35]. Therefore, to treat inflammation and autoimmune diseases effectively, new therapeutic approaches should be considered [36].

## 3. Composition and Immune Modulatory Function of Extracellular Vesicles 

Extracellular vesicles (EVs) are small and heterogenous endosome-derived vesicles encased in a phospholipid bilayer. These vesicles emerge from most cells and exist in body fluids including the blood, bile, urine, and saliva [5]. EVs have been divided into three types by their mode of biogenesis, cargo composition, and release pathway: apoptotic bodies (with diameters >1000 nm), microvesicles (100–1000 nm), and exosomes (30–150 nm) [37]. On the basis of their size, EVs are further divided into small (<100 nm) and medium/large (>200 nm) categories [38]. The terms “small EV” and “exosome” are used interchangeably in many scientific papers. Exosomes are produced through the endosomal system in a pathway that involves sequential formation of the early endosome, late endosome, and multivesicular body (MVB), where intraluminal vesicles (ILVs) are generated through intraluminal budding. When MVBs fuse with the plasma membrane, ILVs are released extracellularly; these released ILVs are called exosomes [39]. Because exosomes originate from the endosome, they contain various biomolecular constituents according to their cell of origin. Therefore, exosomes have been described as a “fingerprint” of the origin cell [40].

Exosomes contain nucleic acids, metabolites, lipids, proteins, and peptides from the cell of origin [41]. The exosome membrane contains numerous raft-associated lipids, including cholesterol, sphingolipids, ceramide, glycerophospholipids, phosphatidylcholine, and phosphatidylserine [42]. The exosomal membrane also contains various members of the tetraspanin family of membrane proteins, including CD63, CD81 and CD9, heat shock proteins, adhesion molecules, integrins, and ESCRT-related proteins such as Alix and TSG101. Because of their abundance, tetraspanins, Alix, TSG101, and HSP70 are used as exosome markers [43]. Exosomes also contain a variety of RNAs, such as small RNAs, long RNAs, coding RNAs, non-coding RNAs, mRNAs, and miRNAs [44].

Exosomes mediate horizontal cell–cell communication through the activity of their intraluminal biomolecules, including proteins, nucleic acids, and metabolites. These molecules are taken up by recipient cells through phagocytosis, micropinocytosis, clathrin-mediated endocytosis, caveolin-dependent endocytosis, and plasma membrane fusion, after which they are involved with intracellular signaling [39]. When RNAs are delivered via fusion of exosomes with recipient cells, they play a role in regulating many aspects of the cell’s phenotype and behavior, including the cell cycle, propensity for apoptosis, migration, inflammation, and angiogenesis [45].

Because they reflect information about the cell of origin, exosomes have the potential to serve as disease biomarkers. For example, the exosomes produced by cancerous tissue are not only abundant, but also include multiple cancer markers shed by cancer cells [46]. Tumor antigens obtained directly from cancerous tissue or from blood tests may be present at much lower concentration. Furthermore, the identification of cancer in organs that are not exposed externally can involve invasive procedures, but exosomes can be obtained through non-invasive means. Therefore, exosomes may be very useful as highly sensitive and non-invasive markers for both the diagnosis and prognosis of cancer [47].

Exosomes’ reflection of the properties and characteristics of the origin cell also has value in the realm of cell therapy. Problems in this area can potentially be solved by using exosomes as a cell substitute. Exosomes derived from certain cell types exhibit therapeutic effects in and of themselves. The source of exosomes with therapeutic effect comes from various sources as well as cell culture media. For example, immune cell- or mesenchymal stem cell (MSC)-derived exosomes contain biomolecules that can affect the proliferation or activity of the recipient’s innate and adaptive immune systems and thus function in immune regulation [5]. Exosomes extracted from milk are absorbed in the human gastrointestinal tract in an intact form, can be delivered orally, and are expected to be used as a drug delivery system for the treatment of various diseases through the advantage of effectively delivering chemotherapy agents [48,49]. Blood exosomes are used as biomarkers to diagnose diseases or to monitor progression or treatment efficacy [50,51]. Urine exosomes can be used as a biomarker to identify the kidney and genitourinary tract, and when injected, urinary exosomes migrate to the kidney. They aid tubular cell proliferation, and reduce inflammatory markers, resulting in renal recovery [52,53].

In addition to their innate effects, exosomes are also promising as natural drug loading and delivery platforms. Exosomes exhibit structural stability and desired cargos can be loaded through physical, chemical, or biological approaches [54]. The lipid bilayer protects contents from enzymatic (including RNase) degradation, minimizing the loss of internal biomolecules or drugs [7]. Exosomes can be administered through intranasal, intravenous, intraperitoneal, and intracranial routes [55,56]. They can pass through the blood–brain barrier, providing a means to deliver drugs to the brain [57]. There are several types of synthetic nanocarriers for drug delivery, such as liposomes, dendrimers, and polymers. However, because these carriers are synthetic, they could be immunogenic when they enter the body [58]. Exosomes provide a non-toxic alternative. Exosomes are less immunogenic, and exhibit improved pharmacokinetic and pharmacodynamic properties compared to synthetic nanocarriers [6]. Because of these advantages, the use of exosomes as a drug delivery system is an active area of research. For example, catalase, which has antioxidant effects, exhibited significant neuroprotective effects when loaded into exosomes in vitro, and in an in vivo Parkinson’s disease model [59]. Exosomes loaded with cisplatin derived from umbilical cord macrophages have increased cytotoxicity in human ovarian cancer cell lines [60]. Bovine milk-derived exosomes loaded with withaferin A exhibited tumor-suppressing effects in mice bearing human lung cancer xenografts [61]. In a LPS induced septic shock mouse model, curcumin-loaded mouse lymphoma cell-derived exosomes enhanced apoptosis of CD11b^+^ Gr-1^+^ myeloid cells, triggering anti-inflammatory activity [62]. Exosomes loaded with dexamethasone exhibited anti-inflammatory effects in RAW264.7 cells in vitro. The exosomes preserved bone and cartilage, and reduced joint inflammation in a collagen induced arthritis (CIA) mouse model [63]. Doxorubicin encapsulated exosomes derived from mouse immature dendritic cells reduced breast cancer cell viability in vitro and inhibited tumor growth in a tumor-bearing nude mouse model without toxicity [64].

In the next section, we will review various inflammatory diseases and therapeutic applications of exosomes for such diseases. 

## 4. Therapeutic Effects of EVs on Inflammatory Diseases

**Figure 3:**
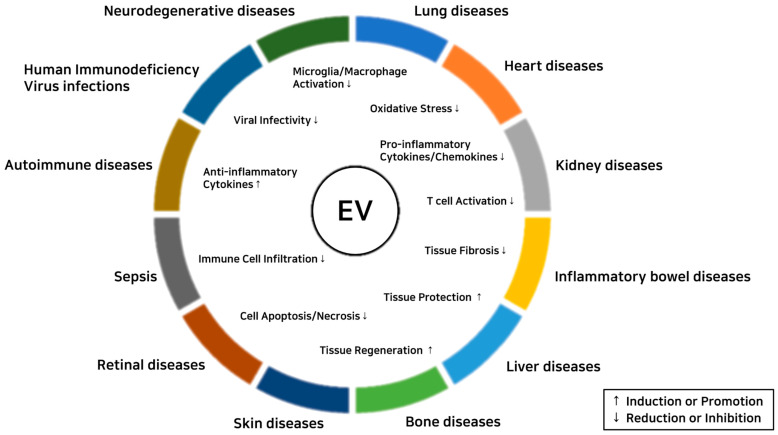
Therapeutic effects of extracellular vesicles (EV) on inflammatory diseases.

EVs show immunomodulatory and tissue-protective effects on inflammatory diseases (Figure 3). Therapeutic effects of EVs on each inflammatory disease are summarized below.

### 4.1. Neurodegenerative Diseases

Neurodegenerative diseases involve the loss of functional neurons as a result of many different causes. Acute or chronic neuroinflammation is an important factor in the pathogenesis of such diseases [65,66], which have been a primary target of studies involving EV therapy.

Therapeutic effects of MSC-derived EVs on neuroinflammation have been reported [67,68,69]. Ding et al. observed that human umbilical cord MSC (UC-MSC)-derived exosomes attenuated neuroinflammation in vitro and in vivo in BV2 microglial cells and the APP/PS1 mouse model of Alzheimer’s disease, respectively [70]. Losurdo and colleagues showed that human bone marrow MSC (BM-MSC)-derived EVs inhibited the activation of primary murine microglia in vitro and in vivo in the 3xTg mouse model of Alzheimer’s disease [71]. A recent study reported that human adipocyte MSC (AD-MSC)-derived EVs alleviated amyloid β_1-42_-induced activation of microglia in the APP/PS1 mouse model [71]. Several other studies have investigated the anti-inflammatory effects of EVs on traumatic brain injury (TBI) or perinatal brain injury (PBI) [72,73,74]. Ni et al. showed that EVs from rat BM-MSCs inhibited inflammation at an early stage of TBI in vivo by polarizing M1 macrophages to the anti-inflammatory M2 phenotype [72]. Another recent study reported that miRNA (miR)-711-loaded EVs from the BV2 cell line induced M2 polarization of microglia and the production of the anti-inflammatory cytokine IL-10 in vitro. These EVs also decreased the level of TNF-α, increased the level of IL-10, and promoted M2 polarization of microglia in the controlled cortical impact-induced TBI mouse model [71]. In other experiments, EVs from human Wharton’s jelly MSCs (WJ-MSCs) were found to reduce the expression of inflammatory proteins (CXCL2, CXCL10, IL-1β, IL-18, and TNF-α) and activation of microglia in a PBI rat model [74]. Another study reported that human BM-MSC-derived EVs reduced the activation of ipsilateral microglia in a hypoxia-ischemia induced PBI mouse model [73]. Immunomodulatory and neuro-regenerative effects of MSC-derived exosomes have also been reported in status epilepticus (SE). EVs from intranasal MSCs alleviated inflammation and inhibited abnormal neurogenesis in a mouse model of SE [75]. Another study showed that human UC-MSC-derived EVs dampen LPS-induced activation of astrocytes by reducing astrogliosis and NF-κB expression in the SE mouse model [76]. 

Therapeutic effects of EVs on spinal cord injury (SCI) have also been investigated. In a recent study, Jiang et al. reported suppressive effects of neuron-derived exosomal miR-124-3p on activated microglia and astrocytes in a traumatic SCI mouse model [77]. Liu et al. investigated the effects of EVs from hypoxia-conditioned human BM-MSCs, which are enriched with miR-216a-5p, and showed that exosomal miR-216a-5p promoted M1 to M2 polarization of microglia in the traumatic SCI mouse model [78]. Neural stem cell (NSC)-derived small EVs also exhibited anti-apoptotic and anti-inflammatory effects by inducing autophagy in a traumatic SCI rat model [79]. Human UC-MSC-derived EVs attenuated inflammation by inducing M2 polarization of M1 macrophages, resulting in functional recovery in an SCI mouse model [80]. Recently, Huang et al. reported that EVs from epidural fat-MSCs (EF-MSCs) decreased activation of the NLRP3 inflammasome and promoted functional recovery in an SCI rat model [81].

### 4.2. Lung Diseases

Acute lung inflammation results from an innate immune defense against invading microbes; chronic inflammation can occur when the response fails to remove the inflammatory stimulus [82]. Inoculation of a mouse model of traumatic acute lung injury (ALI) with BM-MSC-derived EVs suppressed the immune response and reduced oxidative stress [83]. The production of TNF-α, IL-6, and IL-8 in the EV-treated mice was significantly reduced, and the number of inflammatory cells was decreased, compared to that in untreated ALI-induced mice. Furthermore, superoxide dismutase activity in lung tissue increased, and levels of malondialdehyde and H_2_O_2_, markers of oxidative stress, decreased. These effects were possibly due to P2X ligand-gated ion channel 7 (P2 × 7) silencing, a downstream effect of exosomal miR-124-3p [83]. In another study, EVs isolated from miRNA-30b-3p overexpressing MSCs showed protective effects in mice with LPS-induced ALI [84]. Production of serum amyloid A3, which is secreted during acute inflammation, was reduced in the EV-treated mice, indicating an inhibitory role for exosomal miR-30b-3p. Furthermore, pro-inflammatory cytokines (IL-1β, TNF-α, and IL-6) were downregulated, whereas the anti-inflammatory cytokine IL-10 was upregulated, in mouse lung tissues [84]. 

In other work, EVs from induced pluripotent stem cell (iPSC)-derived MSCs showed immunomodulatory effects in a mouse model of allergic airway inflammation [85]. In these MSC-derived EV-treated mice, the number of eosinophils decreased dramatically, whereas the number of neutrophils remained unchanged, compared to untreated mice. Cytokines (IL-4, IL-5, and IL-13) related to Type 2 helper T cells, which contribute to allergic responses, were significantly reduced in bronchoalveolar lavage (BAL) fluid from the treated mice [85]. In experiments involving human cells, it has been shown that MSC-derived EVs have an important role in enhancing Treg proliferation [86]. Treatment of EVs to peripheral blood mononuclear cells (PBMCs) derived from asthmatic patients led to elevated IL-10 and TGF-β expression levels, which promote Treg (CD4^+^ CD25^+^ FoxP3^+^ T cells) function and proliferation [86]. Another research group reported that EVs derived from interferon-γ (IFN-γ) preconditioned MSCs attenuated *Escherichia coli*-induced pneumonia in rats [87]. These authors found that the alveolar TNF-α concentration was reduced in BAL fluid from rats with pneumonia treated with such EVs. Additionally, endothelial nitric oxide synthase (eNOS) expression was restored in the lung tissues of the EV-treated rats; this expression level was significantly higher than that in either the PBS-treated group or the group treated with EVs derived from naïve MSCs [87]. 

### 4.3. Heart Diseases 

Regarding heart disease, the therapeutic effects of EVs have mainly been tested in models of acute myocardial infarction (AMI). In AMI, myocardial cells die in an acute manner because of blockage of coronary vessels and poor blood flow that results in persistent ischemia [88]. This is one of the leading causes of death in the West [89]. Administration of BM-MSC-derived exosomes to mice with induced myocardial infarction (MI) has been shown to reduce the infiltration of inflammatory cells [90,91,92,93]. Blood levels of pro-inflammatory cytokines (TNF-α, IL-6) were reduced at 3 and 7 days after administration of the exosomes [90]. In ischemic heart regions, recruited macrophages showed upregulated levels of an M2 macrophage marker (CD206), but downregulated levels of an M1 marker (CD11c). At the same time, it was observed that the levels of Bax and cleaved caspase-3, as well as cardiomyocyte apoptosis, were reduced in the infarcted heart. These results indicate that MSC-derived EVs relieve inflammation and restore function in the MI heart [90]. Other experiments showed that treatment with BM-MSC-derived EVs improved the echocardiographic performance of infarcted hearts in mice and reduced fibrosis in the heart [91]. Furthermore, BM-MSC-derived EVs were found to significantly reduce the number of infiltrating CD68^+^ cells and reduce infarct size in a rat model of MI [92].

MiR-93-5p containing EVs derived from AD-MSCs have immune regulatory effects on MI model mice and AMI patients [94]. Such EVs reduced the level of TLR4 expression and NF-κB phosphorylation in myocardial tissue, resulting in downregulation of pro-inflammatory cytokines (IL-6, IL-1β, and TNF-α) in blood samples from AMI patients. Therefore, it was suggested that exosomal miR-93-5p prevents myocardial injury caused by TLR4-mediated inflammatory responses and Atg7-mediated autophagy, which is a known target of miR-93-5p [94]. 

In an elastase-induced abdominal aortic aneurysm mouse model, MSC-derived EVs reduced aortic levels of pro-inflammatory cytokines (INF-γ, IL-17, IL-23, TNF-α, and MCP-1) and infiltration of inflammatory cells into aortic tissue [93]. This immune modulatory effect is caused by miR-147.

### 4.4. Kidney Diseases

Kidney injury is accompanied by structural damage and loss of function, and causes a variety of symptoms such as sepsis, nephrotoxicity, and ischemia in patients [95]. Acute kidney injury (AKI) is the most frequently studied renal disease. Several groups have reported that EVs promote renoprotection in AKI model mice [96,97,98]. UC-MSC-derived EVs have immune modulatory effects; they increase the frequency of Tregs (CD4^+^CD25^+^FOXP3^+^ T cells) while suppressing T-cell proliferation, leading to an improvement in acute tubular necrosis in the AKI-induced mice [96]. Injection of BM-MSC-derived EVs into AKI disease model mice led to reduced tubular lesions [97]. Recently, iPSC-derived EVs were found to promote renoprotection in a mouse model of AKI, which was induced by ischemia-reperfusion injury (IRI) [98]. The expression level of CD206, the M2 macrophage marker, was upregulated compared to its level in IRI mice that were not treated with EVs. iPSC-derived EVs also reduced oxidative stress in renal tissues, which exhibited increased expression levels of SOD1, aldehyde oxidase-1 (AOX1), sirtuin1 (SIRT1), and sirtuin2 (SIRT2), but reduced IRI-induced expression of inducible nitric oxide synthase (iNOS). These findings indicate that iPSC-derived EVs have immune regulatory and anti-oxidative functions [98]. 

In a swine model of metabolic syndrome and renal artery stenosis, AD-MSC-derived EVs were shown to reduce stenotic kidney inflammation and restore renal function [99]. Immunostaining of the stenotic kidney revealed that the M1/M2 phase macrophage ratio decreased; it was confirmed that EVs caused the polarization of macrophages to the M2 phase. In addition, levels of proinflammatory cytokines (TNF-α, IL-6, and IL-1β) decreased in the renal vein, but levels of the anti-inflammatory cytokine IL-10 increased. The immune modulatory effects disappeared when IL-10-depleted EVs were used, suggesting that IL-10 plays an important role in immune regulation caused by MSC-derived EVs [99]. 

In a clinical study involving 40 patients, the administration of two doses of UC-MSC-derived EVs was found to have sustained effects (lasting 3–6 months) in ameliorating chronic kidney disease [100]. An increase in the patients’ IL-10 and TGF-β plasma levels was apparent at week 4 after treatment; at week 12, IL-10 exhibited a 3-fold increase and TGF-β a 5-fold increase. The levels gradually decreased from week 12 until month 6, but were still higher at that time than before treatment. Suppression of inflammation allowed recovery of kidney function, as shown by the expression of CD133 and Ki-67, which indicate active recovery of renal tubular cells and active proliferation of tubular epithelial cells, respectively [100]. 

In a study of renal fibrosis induced by unilateral ureteral obstruction in rats, intravenously administered EVs from placenta-derived-MSCs reduced kidney inflammation and fibrosis by promoting CD4^+^ T cell polarization to the Treg phenotype [101]. The PD-MSC-derived EV-treated group, similar to the PD-MSC-treated group, exhibited increased Foxp3 and IL-17 expression in renal tissues.

### 4.5. Inflammatory Bowel Diseases

Inflammatory bowel disease (IBD) represents two chronic intestinal disorders: ulcerative colitis and Crohn’s disease [102]. IBD has become one of the largest disease burdens in newly industrialized countries [103]. Several groups have found that MSC-derived EVs relieved symptoms in dextran sulfate sodium (DSS)-induced IBD model mice [104,105,106]. The body weight, which normally declines in DSS-treated mice, was maintained for 10 days after the administration of UC-MSC-derived EVs, and the colon length, which was shortened by DSS treatment, was restored (64 to 77 mm) [104]. In addition to this effect, structural damage to the villi caused by intestinal inflammation was alleviated, and expression levels of pro-inflammatory cytokines (IL-1β and IL-6) were observed to be significantly reduced, whereas levels of the anti-inflammatory cytokine IL-10 were increased. Neddylation, a process in which the ubiquitin-like protein NEDD8 is covalently attached to a target protein as a post-translational modification, is proposed to be associated with IBD pathogenesis mediated by dendritic cells (DCs) [107]. EV-treated mice showed reduced levels of neddylation-related molecules (cullin 1 and NEDD8) in colon tissues [104]. MiR-326 was suggested as an important regulator, in part because of a potential binding site in the 3′ untranslated region of the NEDD8 mRNA. Thus, exosomal miR-326 may relieve IBD by inhibiting neddylation [104]. EVs also reduced levels of increased JAK1 and STAT1 phosphorylation caused by DSS-induction in the colon tissues of the mouse model. These findings suggest that BM-MSC-derived EVs might mediate immune responses by the JAK-STAT signaling pathway [105]. Furthermore, UC-MSC-derived EVs reduced levels of the pro-inflammatory cytokine IL-7 in colon tissues from both colitis model mice and human patients [106].

Further studies have been done in a rat model in which colitis was induced by 2,4,6-trinitrobezenesulfonic acid. Administration of EVs, derived from BM-MSCs that overexpressed the anti-inflammatory miR-146a, was found to have immunomodulatory effects, suppressing inflammatory cytokines (TNF-α, IL-6, and IL-1β) in colon tissue [108]. MiR-146a targets TNF receptor-associated factor 6 (TRAF6) and IL-1 receptor-associated kinase 1 (IRAK1), resulting in inhibition of the NF-κB signaling pathway. 

### 4.6. Liver Diseases

Liver fibrosis and cirrhosis are lethal diseases that result from chronic liver inflammation [109], and EVs have been considered as therapeutic agents for such diseases [110,111]. EVs from human amnion-derived MSCs (AM-MSCs) significantly reduced expression of inflammatory cytokines (TNF-α, IL-1β, IL-6, MCP-1, and TGF-β) and M1 macrophage marker proteins (CD68 and CD11c) in the liver tissue of rats in which nonalcoholic steatohepatitis (NASH) had been induced by a high-fat diet (HFD). AM-MSC-derived EVs also decreased LPS-induced inflammation by down-regulating TNF-α, IL-1β, IL-6, and MCP-1 expression in Kupffer cells and TNF-α expression in hepatic stellate cells in vitro [112]. Another study reported that human UC perivascular cell-derived EVs also reduced hepatic stellate cell activation. Furthermore, the EVs modulated the polarization of M1 hepatic macrophages to an anti-inflammatory phenotype in vitro by decreasing expression of iNOS, arginase-1, IL-6, and TNF-α [113]. Li et al. have reported anti-inflammatory and anti-fibrosis effects of hepatocyte-derived EVs. The EVs alleviated liver fibrosis and inflammation by decreasing expression of fibrosis-related proteins (alpha smooth muscle actin (α-SMA), collagen 1a1 (Col1a1), and cellular communication network factor (CCN2)) and inflammatory chemokines ((chemokine (C-C motif) ligand 3 (CCL3), CCL5, CCL12, tissue inhibitor of metallopeptidase-1 (TIMP-1), and triggering receptor expressed on myeloid cells-1 (TREM-1)) in a CCl4-induced hepatic fibrosis mouse model [114]. A recent study showed that human liver stem cell-derived EVs attenuated liver fibrosis and inflammation in a mouse model, in which NASH was induced by a methionine- and choline-deprived diet, by modulating pro-fibrotic and pro-inflammatory gene expression [115]. 

EVs also have therapeutic effects on acute or chronic liver injury [116,117,118,119,120]. EVs from human embryonic stem cell-derived MSCs caused immunomodulatory effects by decreasing the gene expression of pro-inflammatory cytokines (TNF-α and IL-2) and by increasing the gene expression of anti-inflammatory cytokines (TGF-β1 and IL-10) in liver tissues in a thioacetamide-induced chronic liver injury rat model [119]. Human UC-MSC-derived EVs were found to inhibit the infiltration of neutrophils into the liver and reduce liver tissue inflammation in a hepatic IRI rat model [120]. Human AD-MSC-EVs also caused anti-inflammatory effects by reducing the levels of several kinds of inflammation-related cytokines and chemokines in an acute liver failure rat model [117]. Recently, Kawata et al. reported that EVs from RAW 264.7 cells pretreated with concanavalin A induced anti-inflammatory effects by reducing the production of inflammatory cytokines (IL-6, IL-1β, and TNF-α) in a concanavalin A-induced hepatitis mouse model [118]. Chen et al. investigated the protective effects of mouse BM-MSC-derived EVs. They observed reduced expression of inflammation-related cytokines (TNF-α, IL-17, and IL-1β) in liver tissues in a mouse model (in which autoimmune hepatitis was induced by the liver antigen S100) in an exosomal miR-223-dependent manner. This treatment also inhibited NLRP3 inflammasome activation [116].

### 4.7. Bone Diseases

Osteoarthritis (OA) involves local inflammation of the synovium, which lead to loss of cartilage [121]. EVs have emerged as a potential agent for treating inflammation-induced bone diseases [122,123]. EVs from human AD-MSCs increased expression of the anti-inflammatory cytokine IL-10 and reduced expression of IL-1β-induced inflammatory cytokines (IL-6, TNF-α, and PGE_2_) by osteoarthritic osteoblasts in vitro [124]. Zhang et al. collected EVs from human embryonic stem cell-derived MSCs and showed that the EVs alleviated IL-1β-induced inflammation of condylar chondrocytes by reducing NO and matrix metalloproteinase 13 (MMP13) production [125]. Vonk et al. reported that EVs from human BM-MSCs caused anti-inflammatory effects by modulating TNF-α-induced expression of cyclooxygenase 2 (COX2), IL-1α, IL-1β, IL-6, IL-8, IL-17, and NF-κB by chondrocytes from OA patients [126]. Another study reported that human AD-MSC-derived small EVs reduced IL-1β-induced production of MMP-1, MMP-3, and MMP-13 by chondrocytes from OA patients. The EVs also inhibited M1 macrophage infiltration and reduced IL-1β-positive cells in the synovium [127]. A recent study showed that EVs from rat BM-MSCs reduced MMP-13 expression in rat chondrocytes and reduced serum levels of IL-1β, IL-6, and TNF-α in vivo [128].

### 4.8. Skin Diseases

Inappropriate responses by cells of the skin immune system can cause chronic skin diseases [129]. Cho et al. reported that EVs from human AD-MSCs decreased serum levels of pro-inflammatory cytokines (IL-4, IL-31, IL-23, and TNF-α) in the NC/Nga mouse model of atopic dermatitis. The serum IgE level and the number of eosinophils also decreased [130]. In another study, AD-MSC-derived EVs showed anti-inflammatory effects in the hairless SKH-1 mouse model in which atopic dermatitis was induced. EVs decreased the levels of IL-4, IL-5, IL-13, TNF-α, IFN-γ, IL-17, IgE, and thymic stromal lymphopoietin [131]. EVs have also been derived from prokaryotic cells, including the probiotic bacterium *Lactobacillus plantarum*. Recently, such EVs were shown to promote polarization of THP-1 cells to macrophages that have an M2b phenotype in vitro. The EVs also induced secretion of IL-10 by human skin organ cultures, suggesting that *Lactobacillus plantarum*-derived EVs have therapeutic potential in skin inflammation [132]. Moreover, human BM-MSC- and human jaw BM-MSC-derived EVs promoted M2 polarization of human monocytes in vitro and accelerated cutaneous wound healing in vivo [133].

### 4.9. Retinal Diseases

Inflammation disrupts retinal homeostasis, which can cause severe loss of vision [134]. EVs have immunomodulatory and anti-fibrotic effects in retinal disease [135,136]. In a rat retinal ischemia model, EVs from human BM-MSCs decreased the levels of TNF-α, IL-6, and cleaved caspase 3 in retinal tissues [137]. Another study reported that corneal stromal stem cell-derived MSCs secrete exosomes, which reduce neutrophil infiltration at corneal wound sites through regulation of myeloperoxidase expression [138]. Tal et al. reported that EVs from human PD-MSCs have anti-inflammatory effects on human corneal epithelial cells by reducing expression of pro-inflammatory cytokines (IL-1β, TNF-α, IL-8, and NF-κB) but promoting expression of the anti-inflammatory cytokine IL-10 [139]. A recent study showed that human AD-MSC-derived EVs exerted suppressive effects on NLRP3 inflammatory responses by reducing expression of inflammatory cytokines (IL-1β and IL-18) and of NLRP3 inflammasome subunits (NLRP3, ASC, and caspase 1) in a human corneal epithelial cell line [140].

### 4.10. Sepsis

Sepsis, a life-threatening condition, results from a dysregulated response to infection. It affects 20 million patients worldwide each year and accounts for a large portion of the mortality rate [141]. Several studies have found that MSC-derived EVs lowered systemic inflammation and alleviated organ damage in sepsis [142,143,144]. EVs from healthy AD-MSCs reduced the circulating level of TNF-α, and this led to a significant increase in the survival rate in a rat model of sepsis [142]. Another group examined the effect of AD-MSC-derived EVs on sepsis in mice. They administered AD-MSC-derived EVs and observed regulation of LPS-induced inflammation via the Notch-miR-148a-3p axis and consequent relief of sepsis [143]. In a recent study, super-repressor IκB (srIκB, a dominant active form of IκBα) was loaded into EVs, and the effect on sepsis-associated organ damage and mortality was observed [144]. srIκB-loaded EVs improved the survival rate of mice in which sepsis was induced by LPS or cecal ligation and puncture.

### 4.11. Autoimmune Diseases

An autoimmune state can cause systemic inflammation as well as the functional loss of specific organs, which result in several types of autoimmune disease [145]. Studies have reported that EVs are involved in the development or attenuation of autoimmune disease [146]. MSC-derived EVs are emerging as a therapeutic agent to treat autoimmune related-inflammation through their immunomodulatory effects [147,148]. Human UC-MSC-derived EVs were found to reduce the production of inflammatory cytokines (IL-2, TNF-α, and IFN-γ) and promote anti-inflammatory cytokine IL-10 production in Concanavalin A-activated splenocytes and in graft-versus-host disease (GVHD) in a mouse model of allogeneic hematopoietic stem cell transplantation. In addition, UC-MSC-derived EVs suppressed and altered GVHD-induced CD4^+^ and CD8^+^ T cell phenotypes [149]. Another study also reported a therapeutic effect of human BM-MSC-derived EVs in an acute GVHD mouse model. EVs suppressed CD4^+^ and CD8^+^ T cells and preserved the naïve T cell population in various GVHD-targeted organs [150]. A recent study showed that EVs from human UC-MSCs prevented chronic GVHD-induced skin fibrosis by modulating the activation of macrophages and B cell responses in vivo [151].

Evidence suggests that DCs play a role in type 1 diabetes. One study showed that human BM-MSC-derived EVs induced DCs, which had been differentiated from monocytes from type 1 diabetes patients, to acquire an immature phenotype. When co-cultured with T cells, these DCs suppressed the number of Th 17 cells and the level of IL-17 and IFN-γ, compared to that seen with untreated DCs [152]. Another study also reported that mouse AD-MSC-derived EVs exert anti-inflammatory effects in mice with type 1 diabetes. EVs also decreased the production of pro-inflammatory cytokines (IFN-γ and IL-17) and increased the production of anti-inflammatory cytokines (IL-4, TGF-β, and IL-10) by splenic mononuclear cells in vitro [153].

Rheumatoid arthritis (RA), a systemic and chronic autoimmune disease, lead to joint pain and damage [154]. Recently, therapeutic effects of EVs in RA were reported [155]. Cosenza et al. reported an immunosuppressive effect of mouse BM-MSC-derived EVs in vivo. EVs indirectly inhibited T lymphocyte proliferation in a CIA mouse model. The levels of IL-6, TNF-α, and IL-1β in supernatants from resting lymph nodes also decreased [156]. Another study showed that EVs from rat BM-MSCs reduced the levels of TNF-α, IL-1β, and the inflammatory mediator prostaglandin E2 (PGE_2_) in synovial tissues and serum from the CIA rat model. Plasma NO and iNOS levels also decreased. In this study, exosomal miR-192-5p was identified as a functional molecule directly targeting the Ras-related C3 botulinum toxin substrate 2 gene, which resulted in improvement of the pathophysiology in CIA model rats [157]. In addition, Meng and Qiu reported that human BM-MSC-derived exosomal miR-320a suppressed CXCL9 expression by synoviocytes from RA patients, which resulted in attenuation of synoviocyte activation and RA-induced inflammation [158].

MS and encephalomyelitis are autoimmune disorders that cause neuroinflammation and demyelination in the central nervous system (CNS). Casella et al. reported that IL-4-overexpressing, BV2-derived EVs modulate the polarization of microglia toward an M2 phenotype by increasing the expression of CD206 and arginase-1 in experimental autoimmune encephalomyelitis (EAE), a mouse model of MS [159]. In another study, Riazifar et al. showed that IFN-γ-primed human BM-MSC-derived EVs inhibit the infiltration of macrophages and T cells into the spinal cord of the EAE mouse model. Co-culture of human PBMCs with IFN-γ-primed human BM-MSC-derived EVs increased secretion of the immunosuppressive protein indoleamine 2,3-dioxygenase and decreased secretion of IL-6, IL-12p70, IL-17AF, and IL-22 [160]. Li et al. reported immunomodulatory effects of rat BM-MSC-derived EVs in an EAE rat model, finding that they attenuated neuroinflammation by inducing microglia polarization toward the M2 phenotype. In addition, the production of TNF-α and iNOS decreased and the production of IL-10, TGF-β, and arginase-1 increased [161]. Moreover, a recent study reported that EVs from human UC blood plasma suppressed CD4^+^ and CD8^+^ cell proliferation and decreased IL-2, IL-6, and IFN-γ expression in an EAE mouse model [162].

In work related to other autoimmune diseases, Shigemoto-Kuroda et al. investigated the therapeutic effects of human MSC-derived EVs in vivo. These authors found that the EVs significantly reduced the expression of genes encoding inflammatory cytokines (IFN-γ, IL-17a, IL-2, IL-1β, IL-6, and IL-12a) and suppressed the development of Th1 and Th17 cells in an experimental autoimmune uveoretinitis mouse model [163]. Furthermore, Wang et al. showed that EVs derived from rat BM-MSCs overexpressing the anti-aging gene Klotho attenuated inflammation by reducing the expression of IL-6 and TNF-α in an acute pancreatitis model [164].

### 4.12. Human Immunodeficiency Virus (HIV) Infections

Prolonged inflammatory state in HIV patients causes chronic activation of innate and adaptive immune systems, leading to multiple organ morbidity [165]. Several studies suggesed that various sources of exosomes have anti-HIV activity [166,167,168]. One research group tested whether semen exosomes from healthy donors can inhibit replication of HIV-1 [166]. The semen exosomes were pre-incubated with target cells for 1 h prior to infection, and the levels of HIV-1 DNA and RNA were quantified. The results showed reduced infectivity in various cell types and was comparable to the effects of azidothymidine (AZT) [166]. Another group showed that breast milk-derived exosomes from healthy donors block HIV-1 transfer from monocyte-derived dendritic cells (MDDCs) to CD4^+^ T cells as well as inhibiting HIV-1 infection of MDDCs [167]. MDDCs were pre-incubated with milk exosomes for 1 h, and HIV-1 infected MDDCs were cocultured with CD4^+^ T cells for 5 days. The percentage of p24^+^ in CD4^+^ T cells significantly diminished, indicating reduced productive infection [167]. One research group in Brazil showed CD4^+^ T cell-derived exosomes significantly reduced the HIV-1 infectivity by hindering the interaction between virus and target cells [168].

### 4.13. Other Inflammation-Related Conditions

One of the most important changes that occurs during the aging process is the dysregulation of the immune response, which can lead to a chronic and systemic inflammatory state [169]. There are ongoing investigations that aim to alleviate this so-called inflammaging [170,171]. One research group utilized EVs containing miR-192 to relieve age-related inflammation [170]. They found that the level of miR-192 was higher in serum EVs from aged mice (14–18 months) than in those from young mice (2–3 month) due to a hyperinflammatory state in aged mice. This phenomenon was associated with higher serum IL-6 levels in aged mice compared to young mice. Surprisingly, miR-192-containing EVs attenuated IL-6 and CCL2 expression in RAW 264.7 cells and human macrophages (derived from CD14^+^ monocytes) stimulated by either CL097 or R848, which are synthetic TLR7 ligands. This phenomenon is due to a mechanism in which exosomal miR-192 relieves excessive inflammation through a negative feedback loop in aged mice [170]. There has been an attempt to rejuvenate aged mice with EVs extracted from serum from young mice [171]. The expression level of pro-inflammatory cytokines (IL-6, IL-1β, and TNF-α) and the number of CD4^+^IFN-γ^+^ T cells in serum from old mice significantly decreased after treatment with such EVs. In the CNS of aged mice, the percentages of CD45^+^CD11b^-^CD3^+^ cells, CD45^high^CD11b^+^ macrophages, and CD45^-/low^CD11b^+^ microglia were reduced after injection of EVs derived from young mice. Importantly, aging-related thymic phenotypes were attenuated in old mice after administration of EVs. In addition to recovery of the thymus size and microstructure in aged mice, there was an increase in molecules associated with negative selection during T cell development, including Aire and Nur77 [171].

Regarding obesity, excess nutrients cause adipose tissue to secrete inflammatory factors such as TNF-α and IL-6; inflammation is associated with cardiovascular disease, atherosclerosis, metabolic syndrome, and insulin resistance. The liver synthesizes C-reactive protein, a marker of inflammation, due to such stimulation [172]. Some research groups have utilized EVs and found that they reduced adipocyte inflammation by promoting M2 phase polarization in macrophages [173,174]. When HFD-fed mice were treated with AD-MSC-derived EVs, white adipose tissue (WAT) inflammation was alleviated; the expression level of pro-inflammatory cytokines (TNF-α, IL-12, and IL-6) was reduced and that of the anti-inflammatory cytokine IL-10 was increased [173]. Moreover, the expression level of arginase-1 (an M2 phase macrophage marker) was significantly increased by active STAT3. M2 macrophages promote AD-MSC proliferation and increase the expression of tyrosine hydroxylase (TH). TH and lactate produced by AD-MSCs increase the expression of uncoupling protein 1 (UCP1) in WAT, which promotes beiging [173]. Another group reported that miR-34a contained in adipocyte-derived exosomes suppressed M2 phase macrophage polarization in obesity-induced adipose tissues via downregulation of Krüppel-like factor 4 (Klf4) [174]. Exosomal miR-34a protected obese mice from glucose intolerance and insulin resistance.

EVs may have a role in suppressing allergic reactions. In one recent report, exosomal miR-150 was shown to have a suppressive effect in a mouse model of delayed-type hypersensitivity to casein [175]. 

## 5. Conclusions

Inflammation is involved in the pathogenesis of many diseases; acute or chronic immune responses in target organs can cause functional organ loss. The number of trials studying the therapeutic potential of native or engineered EVs for treating inflammatory diseases is increasing [176,177]. Beginning in 2014, there has been an official movement to establish a guide for the harvest, isolation, characterization, and functional testing of EVs; the guide is now being used as a standard for EV researchers [38,178]. However, setting standards for purity, specific markers, active pharmaceutical ingredients, CMC (chemistry, manufacturing and controls) for manufacturing, and minimizing variation between batches of isolated EVs are issues that remain to be resolved. To circumvent these limitations, many trials for obtaining EVs with robust therapeutic activity and purity are being extensively carried out. Combinations of and comparisons between several EV isolation methods [179], pre-conditioning (priming) cells with various factors [4], and engineering EVs [180] are all being tested so that EVs that exhibit consistent therapeutic effects can be robustly harvested. For clinical applications, the development of methods for producing EVs in compliance with Good Manufacturing Practice is also in progress [181]. In conclusion, EVs have great potential for treating a wide variety of inflammatory diseases. For clinical applications, future efforts will be focused on the manufacturing process, characterization of EVs, and EV-associated safety issues.

## References

[1] Okin D., Medzhitov R. (2012). Evolution of inflammatory diseases. Curr. Biol..

[2] Schett G., Neurath M.F. (2018). Resolution of chronic inflammatory disease: Universal and tissue-specific concepts. Nat. Commun..

[3] Dominguez-Villar M., Hafler D.A. (2018). Regulatory T cells in autoimmune disease. Nat. Immunol..

[4] Joo H.S., Suh J.H., Lee H.J., Bang E.S., Lee J.M. (2020). Current Knowledge and Future Perspectives on Mesenchymal Stem Cell-Derived Exosomes as a New Therapeutic Agent. Int. J. Mol. Sci..

[5] Kalluri R., LeBleu V.S. (2020). The biology, function, and biomedical applications of exosomes. Science.

[6] Morishita M., Takahashi Y., Nishikawa M., Takakura Y. (2017). Pharmacokinetics of Exosomes—An Important Factor for Elucidating the Biological Roles of Exosomes and for the Development of Exosome-Based Therapeutics. J. Pharm. Sci..

[7] Samanta S., Rajasingh S., Drosos N., Zhou Z., Dawn B., Rajasingh J. (2018). Exosomes: New molecular targets of diseases. Acta Pharmacol. Sin..

[8] Newton K., Dixit V.M. (2012). Signaling in innate immunity and inflammation. Cold Spring Harb. Perspect. Biol..

[9] Fullerton J.N., Gilroy D.W. (2016). Resolution of inflammation: A new therapeutic frontier. Nat. Rev. Drug Discov..

[10] Liu T., Zhang L., Joo D., Sun S.C. (2017). NF-kappaB signaling in inflammation. Signal Transduct. Target. Ther..

[11] Turner M.D., Nedjai B., Hurst T., Pennington D.J. (2014). Cytokines and chemokines: At the crossroads of cell signalling and inflammatory disease. Biochim. Biophys. Acta.

[12] Gonzalez-de-Olano D., Alvarez-Twose I. (2018). Mast Cells as Key Players in Allergy and Inflammation. J. Investig. Allergol. Clin. Immunol..

[13] Rondina M.T., Garraud O. (2014). Emerging evidence for platelets as immune and inflammatory effector cells. Front. Immunol..

[14] Yang J., Zhang L., Yu C., Yang X.F., Wang H. (2014). Monocyte and macrophage differentiation: Circulation inflammatory monocyte as biomarker for inflammatory diseases. Biomark. Res..

[15] Oishi Y., Manabe I. (2018). Macrophages in inflammation, repair and regeneration. Int. Immunol..

[16] Kolaczkowska E., Kubes P. (2013). Neutrophil recruitment and function in health and inflammation. Nat. Rev. Immunol..

[17] Watanabe R., Hosgur E., Zhang H., Wen Z., Berry G., Goronzy J.J., Weyand C.M. (2017). Pro-inflammatory and anti-inflammatory T cells in giant cell arteritis. Joint Bone Spine.

[18] Saltiel A.R., Olefsky J.M. (2017). Inflammatory mechanisms linking obesity and metabolic disease. J. Clin. Investig..

[19] Pollack R.M., Donath M.Y., LeRoith D., Leibowitz G. (2016). Anti-inflammatory Agents in the Treatment of Diabetes and Its Vascular Complications. Diabetes Care.

[20] Ferrucci L., Fabbri E. (2018). Inflammageing: Chronic inflammation in ageing, cardiovascular disease, and frailty. Nat. Rev. Cardiol..

[21] Kamada N., Seo S.U., Chen G.Y., Nunez G. (2013). Role of the gut microbiota in immunity and inflammatory disease. Nat. Rev. Immunol..

[22] Brown E.M., Kenny D.J., Xavier R.J. (2019). Gut Microbiota Regulation of T Cells During Inflammation and Autoimmunity. Annu. Rev. Immunol..

[23] Rosenblum M.D., Remedios K.A., Abbas A.K. (2015). Mechanisms of human autoimmunity. J. Clin. Investig..

[24] Zharkova O., Celhar T., Cravens P.D., Satterthwaite A.B., Fairhurst A.M., Davis L.S. (2017). Pathways leading to an immunological disease: Systemic lupus erythematosus. Rheumatology.

[25] Eccleston C., Cooper T.E., Fisher E., Anderson B., Wilkinson N.M. (2017). Non-steroidal anti-inflammatory drugs (NSAIDs) for chronic non-cancer pain in children and adolescents. Cochrane Database Syst. Rev..

[26] Chen Y., Huang J., Tang C., Chen X., Yin Z., Heng B.C., Chen W., Shen W. (2017). Small molecule therapeutics for inflammation-associated chronic musculoskeletal degenerative diseases: Past, present and future. Exp. Cell. Res..

[27] Soualmia F., El Amri C. (2018). Serine protease inhibitors to treat inflammation: A patent review (2011–2016). Expert Opin. Ther. Pat..

[28] Tahamtan A., Teymoori-Rad M., Nakstad B., Salimi V. (2018). Anti-Inflammatory MicroRNAs and Their Potential for Inflammatory Diseases Treatment. Front. Immunol..

[29] Azab A., Nassar A., Azab A.N. (2016). Anti-Inflammatory Activity of Natural Products. Molecules.

[30] Du F.H., Mills E.A., Mao-Draayer Y. (2017). Next-generation anti-CD20 monoclonal antibodies in autoimmune disease treatment. Auto Immun. Highlights.

[31] Phillips B.E., Garciafigueroa Y., Engman C., Trucco M., Giannoukakis N. (2019). Tolerogenic Dendritic Cells and T-Regulatory Cells at the Clinical Trials Crossroad for the Treatment of Autoimmune Disease; Emphasis on Type 1 Diabetes Therapy. Front. Immunol..

[32] Totzke J., Gurbani D., Raphemot R., Hughes P.F., Bodoor K., Carlson D.A., Loiselle D.R., Bera A.K., Eibschutz L.S., Perkins M.M. (2017). Takinib, a Selective TAK1 Inhibitor, Broadens the Therapeutic Efficacy of TNF-alpha Inhibition for Cancer and Autoimmune Disease. Cell Chem. Biol..

[33] Swart J.F., Delemarre E.M., van Wijk F., Boelens J.J., Kuball J., van Laar J.M., Wulffraat N.M. (2017). Haematopoietic stem cell transplantation for autoimmune diseases. Nat. Rev. Rheumatol..

[34] Lu R.M., Hwang Y.C., Liu I.J., Lee C.C., Tsai H.Z., Li H.J., Wu H.C. (2020). Development of therapeutic antibodies for the treatment of diseases. J. Biomed. Sci..

[35] Regmi S., Pathak S., Kim J.O., Yong C.S., Jeong J.H. (2019). Mesenchymal stem cell therapy for the treatment of inflammatory diseases: Challenges, opportunities, and future perspectives. Eur. J. Cell Biol..

[36] Li C., Wang J., Wang Y., Gao H., Wei G., Huang Y., Yu H., Gan Y., Wang Y., Mei L. (2019). Recent progress in drug delivery. Acta Pharm. Sin. B.

[37] Colombo M., Raposo G., Thery C. (2014). Biogenesis, secretion, and intercellular interactions of exosomes and other extracellular vesicles. Annu. Rev. Cell Dev. Biol..

[38] Thery C., Witwer K.W., Aikawa E., Alcaraz M.J., Anderson J.D., Andriantsitohaina R., Antoniou A., Arab T., Archer F., Atkin-Smith G.K. (2018). Minimal information for studies of extracellular vesicles 2018 (MISEV2018): A position statement of the International Society for Extracellular Vesicles and update of the MISEV2014 guidelines. J. Extracell. Vesicles.

[39] Jadli A.S., Ballasy N., Edalat P., Patel V.B. (2020). Inside(sight) of tiny communicator: Exosome biogenesis, secretion, and uptake. Mol. Cell Biochem..

[40] Sancho-Albero M., Navascues N., Mendoza G., Sebastian V., Arruebo M., Martin-Duque P., Santamaria J. (2019). Exosome origin determines cell targeting and the transfer of therapeutic nanoparticles towards target cells. J. Nanobiotechnol..

[41] Mashouri L., Yousefi H., Aref A.R., Ahadi A.M., Molaei F., Alahari S.K. (2019). Exosomes: Composition, biogenesis, and mechanisms in cancer metastasis and drug resistance. Mol. Cancer.

[42] Skotland T., Sagini K., Sandvig K., Llorente A. (2020). An emerging focus on lipids in extracellular vesicles. Adv. Drug Deliv. Rev..

[43] Villarroya-Beltri C., Baixauli F., Gutiérrez-Vázquez C., Sánchez-Madrid F., Mittelbrunn M. (2014). Sorting It out: Regulation of Exosome Loading, Seminars in Cancer Biology.

[44] Dragomir M., Chen B., Calin G.A. (2018). Exosomal lncRNAs as new players in cell-to-cell communication. Transl. Cancer Res..

[45] Maia J., Caja S., Strano Moraes M.C., Couto N., Costa-Silva B. (2018). Exosome-Based Cell-Cell Communication in the Tumor Microenvironment. Front. Cell Dev. Biol..

[46] Wong C.H., Chen Y.C. (2019). Clinical significance of exosomes as potential biomarkers in cancer. World J. Clin. Cases.

[47] LeBleu V.S., Kalluri R. (2020). Exosomes as a Multicomponent Biomarker Platform in Cancer. Trends Cancer.

[48] Betker J.L., Angle B.M., Graner M.W., Anchordoquy T.J. (2019). The potential of exosomes from cow milk for oral delivery. J. Pharm. Sci..

[49] Sedykh S., Kuleshova A., Nevinsky G.J. (2020). Milk Exosomes: Perspective Agents for Anticancer Drug Delivery. Int. J. Mol. Sci..

[50] Sancho-Albero M., Sebastián V., Sesé J., Pazo-Cid R., Mendoza G., Arruebo M., Martín-Duque P., Santamaría J.J. (2020). Isolation of exosomes from whole blood by a new microfluidic device: Proof of concept application in the diagnosis and monitoring of pancreatic cancer. J. Nanobiotechnol..

[51] Abdel-Haq H. (2019). Blood exosomes as a tool for monitoring treatment efficacy and progression of neurodegenerative diseases. Neural Regen. Res..

[52] Grange C., Papadimitriou E., Dimuccio V., Pastorino C., Molina J., O’Kelly R., Niedernhofer L.J., Robbins P.D., Camussi G., Bussolati B.J. (2020). Urinary extracellular vesicles carrying Klotho improve the recovery of renal function in an acute tubular injury model. Mol. Ther..

[53] Street J., Koritzinsky E., Glispie D., Star R., Yuen P. (2017). Urine exosomes: An emerging trove of biomarkers. Advances in Clinical Chemistry.

[54] Fu S., Wang Y., Xia X., Zheng J.C. (2020). Exosome engineering: Current progress in cargo loading and targeted delivery. NanoImpact.

[55] Alvarez-Erviti L., Seow Y., Yin H., Betts C., Lakhal S., Wood M.J. (2011). Delivery of siRNA to the mouse brain by systemic injection of targeted exosomes. Nat. Biotechnol..

[56] Perets N., Hertz S., London M., Offen D. (2018). Intranasal administration of exosomes derived from mesenchymal stem cells ameliorates autistic-like behaviors of BTBR mice. Mol. Autism..

[57] Banks W.A., Sharma P., Bullock K.M., Hansen K.M., Ludwig N., Whiteside T.L. (2020). Transport of Extracellular Vesicles across the Blood-Brain Barrier: Brain Pharmacokinetics and Effects of Inflammation. Int. J. Mol. Sci..

[58] Chinnappan M., Srivastava A., Amreddy N., Razaq M., Pareek V., Ahmed R., Mehta M., Peterson J.E., Munshi A., Ramesh R.J. (2020). Exosomes as drug delivery vehicle and contributor of resistance to anticancer drugs. Cancer Lett..

[59] Haney M.J., Klyachko N.L., Zhao Y., Gupta R., Plotnikova E.G., He Z., Patel T., Piroyan A., Sokolsky M., Kabanov A.V. (2015). Exosomes as drug delivery vehicles for Parkinson’s disease therapy. J. Control. Release.

[60] Zhang X., Liu L., Tang M., Li H., Guo X., Yang X.J., Pharmacy I. (2020). The effects of umbilical cord-derived macrophage exosomes loaded with cisplatin on the growth and drug resistance of ovarian cancer cells. Drug Dev. Ind. Pharm..

[61] Munagala R., Aqil F., Jeyabalan J., Gupta R.C. (2016). Bovine milk-derived exosomes for drug delivery. Cancer Lett..

[62] Sun D., Zhuang X., Xiang X., Liu Y., Zhang S., Liu C., Barnes S., Grizzle W., Miller D., Zhang H.-G. (2010). A novel nanoparticle drug delivery system: The anti-inflammatory activity of curcumin is enhanced when encapsulated in exosomes. Mol. Ther..

[63] Yan F., Zhong Z., Wang Y., Feng Y., Mei Z., Li H., Chen X., Cai L., Li C.J. (2020). Exosome-based biomimetic nanoparticles targeted to inflamed joints for enhanced treatment of rheumatoid arthritis. J. Nanobiotechnol..

[64] Tian Y., Li S., Song J., Ji T., Zhu M., Anderson G.J., Wei J., Nie G.J.B. (2014). A doxorubicin delivery platform using engineered natural membrane vesicle exosomes for targeted tumor therapy. Biomaterials.

[65] Chen W.W., Zhang X., Huang W.J. (2016). Role of neuroinflammation in neurodegenerative diseases (Review). Mol. Med. Rep..

[66] Dugger B.N., Dickson D.W. (2017). Pathology of Neurodegenerative Diseases. Cold Spring Harb. Perspect. Biol..

[67] Dabrowska S., Andrzejewska A., Lukomska B., Janowski M. (2019). Neuroinflammation as a target for treatment of stroke using mesenchymal stem cells and extracellular vesicles. J. Neuroinflamm..

[68] Kalani A., Tyagi A., Tyagi N. (2014). Exosomes: Mediators of neurodegeneration, neuroprotection and therapeutics. Mol. Neurobiol..

[69] Yang Y., Ye Y., Su X., He J., Bai W., He X. (2017). MSCs-Derived Exosomes and Neuroinflammation, Neurogenesis and Therapy of Traumatic Brain Injury. Front. Cell Neurosci..

[70] Ding M., Shen Y., Wang P., Xie Z., Xu S., Zhu Z., Wang Y., Lyu Y., Wang D., Xu L. (2018). Exosomes Isolated From Human Umbilical Cord Mesenchymal Stem Cells Alleviate Neuroinflammation and Reduce Amyloid-Beta Deposition by Modulating Microglial Activation in Alzheimer’s Disease. Neurochem. Res..

[71] Losurdo M., Pedrazzoli M., D’Agostino C., Elia C.A., Massenzio F., Lonati E., Mauri M., Rizzi L., Molteni L., Bresciani E. (2020). Intranasal delivery of mesenchymal stem cell-derived extracellular vesicles exerts immunomodulatory and neuroprotective effects in a 3xTg model of Alzheimer’s disease. Stem Cells Transl. Med..

[72] Ni H., Yang S., Siaw-Debrah F., Hu J., Wu K., He Z., Yang J., Pan S., Lin X., Ye H. (2019). Exosomes Derived From Bone Mesenchymal Stem Cells Ameliorate Early Inflammatory Responses Following Traumatic Brain Injury. Front. Neurosci..

[73] Sisa C., Kholia S., Naylor J., Herrera Sanchez M.B., Bruno S., Deregibus M.C., Camussi G., Inal J.M., Lange S., Hristova M. (2019). Mesenchymal Stromal Cell Derived Extracellular Vesicles Reduce Hypoxia-Ischaemia Induced Perinatal Brain Injury. Front. Physiol..

[74] Thomi G., Surbek D., Haesler V., Joerger-Messerli M., Schoeberlein A. (2019). Exosomes derived from umbilical cord mesenchymal stem cells reduce microglia-mediated neuroinflammation in perinatal brain injury. Stem Cell Res. Ther..

[75] Long Q., Upadhya D., Hattiangady B., Kim D.K., An S.Y., Shuai B., Prockop D.J., Shetty A.K. (2017). Intranasal MSC-derived A1-exosomes ease inflammation, and prevent abnormal neurogenesis and memory dysfunction after status epilepticus. Proc. Natl. Acad. Sci. USA.

[76] Xian P., Hei Y., Wang R., Wang T., Yang J., Li J., Di Z., Liu Z., Baskys A., Liu W. (2019). Mesenchymal stem cell-derived exosomes as a nanotherapeutic agent for amelioration of inflammation-induced astrocyte alterations in mice. Theranostics.

[77] Jiang D., Gong F., Ge X., Lv C., Huang C., Feng S., Zhou Z., Rong Y., Wang J., Ji C. (2020). Neuron-derived exosomes-transmitted miR-124-3p protect traumatically injured spinal cord by suppressing the activation of neurotoxic microglia and astrocytes. J. Nanobiotechnol..

[78] Liu W., Rong Y., Wang J., Zhou Z., Ge X., Ji C., Jiang D., Gong F., Li L., Chen J. (2020). Exosome-shuttled miR-216a-5p from hypoxic preconditioned mesenchymal stem cells repair traumatic spinal cord injury by shifting microglial M1/M2 polarization. J. Neuroinflamm..

[79] Rong Y., Liu W., Wang J., Fan J., Luo Y., Li L., Kong F., Chen J., Tang P., Cai W. (2019). Neural stem cell-derived small extracellular vesicles attenuate apoptosis and neuroinflammation after traumatic spinal cord injury by activating autophagy. Cell Death Dis..

[80] Sun G., Li G., Li D., Huang W., Zhang R., Zhang H., Duan Y., Wang B. (2018). hucMSC derived exosomes promote functional recovery in spinal cord injury mice via attenuating inflammation. Mater. Sci. Eng. C Mater. Biol. Appl..

[81] Huang J.H., Fu C.H., Xu Y., Yin X.M., Cao Y., Lin F.Y. (2020). Extracellular Vesicles Derived from Epidural Fat-Mesenchymal Stem Cells Attenuate NLRP3 Inflammasome Activation and Improve Functional Recovery After Spinal Cord Injury. Neurochem. Res..

[82] Robb C.T., Regan K.H., Dorward D.A., Rossi A.G. (2016). Key mechanisms governing resolution of lung inflammation. Semin. Immunopathol..

[83] Li Q.C., Liang Y., Su Z.B. (2019). Prophylactic treatment with MSC-derived exosomes attenuates traumatic acute lung injury in rats. Am. J. Physiol. Lung Cell. Mol. Physiol..

[84] Yi X., Wei X., Lv H., An Y., Li L., Lu P., Yang Y., Zhang Q., Yi H., Chen G. (2019). Exosomes derived from microRNA-30b-3p-overexpressing mesenchymal stem cells protect against lipopolysaccharide-induced acute lung injury by inhibiting SAA3. Exp. Cell Res..

[85] Fang S.B., Zhang H.Y., Meng X.C., Wang C., He B.X., Peng Y.Q., Xu Z.B., Fan X.L., Wu Z.J., Wu Z.C. (2020). Small extracellular vesicles derived from human MSCs prevent allergic airway inflammation via immunomodulation on pulmonary macrophages. Cell Death Dis..

[86] Du Y.M., Zhuansun Y.X., Chen R., Lin L., Lin Y., Li J.G. (2018). Mesenchymal stem cell exosomes promote immunosuppression of regulatory T cells in asthma. Exp. Cell Res..

[87] Varkouhi A.K., Jerkic M., Ormesher L., Gagnon S., Goyal S., Rabani R., Masterson C., Spring C., Chen P.Z., Gu F.X. (2019). Extracellular Vesicles from Interferon-gamma-primed Human Umbilical Cord Mesenchymal Stromal Cells Reduce Escherichia coli-induced Acute Lung Injury in Rats. Anesthesiology.

[88] Saleh M., Ambrose J.A. (2018). Understanding myocardial infarction. F1000Research.

[89] Jones D.P., Patel J. (2018). Therapeutic Approaches Targeting Inflammation in Cardiovascular Disorders. Biology.

[90] Xu R., Zhang F., Chai R., Zhou W., Hu M., Liu B., Chen X., Liu M., Xu Q., Liu N. (2019). Exosomes derived from pro-inflammatory bone marrow-derived mesenchymal stem cells reduce inflammation and myocardial injury via mediating macrophage polarization. J. Cell. Mol. Med..

[91] Teng X., Chen L., Chen W., Yang J., Yang Z., Shen Z. (2015). Mesenchymal Stem Cell-Derived Exosomes Improve the Microenvironment of Infarcted Myocardium Contributing to Angiogenesis and Anti-Inflammation. Cell. Physiol. Biochem..

[92] Firoozi S., Pahlavan S., Ghanian M.H., Rabbani S., Barekat M., Nazari A., Pakzad M., Shekari F., Hassani S.N., Moslem F. (2020). Mesenchymal stem cell-derived extracellular vesicles alone or in conjunction with a SDKP-conjugated self-assembling peptide improve a rat model of myocardial infarction. Biochem. Biophys. Res. Commun..

[93] Spinosa M., Lu G., Su G., Bontha S.V., Gehrau R., Salmon M.D., Smith J.R., Weiss M.L., Mas V.R., Upchurch G.R. (2018). Human mesenchymal stromal cell-derived extracellular vesicles attenuate aortic aneurysm formation and macrophage activation via microRNA-147. FASEB J..

[94] Liu J., Jiang M., Deng S., Lu J., Huang H., Zhang Y., Gong P., Shen X., Ruan H., Jin M. (2018). miR-93-5p-Containing Exosomes Treatment Attenuates Acute Myocardial Infarction-Induced Myocardial Damage. Mol. Ther. Nucleic Acids.

[95] Makris K., Spanou L. (2016). Acute Kidney Injury: Definition, Pathophysiology and Clinical Phenotypes. Clin. Biochem. Rev..

[96] Kilpinen L., Impola U., Sankkila L., Ritamo I., Aatonen M., Kilpinen S., Tuimala J., Valmu L., Levijoki J., Finckenberg P. (2013). Extracellular membrane vesicles from umbilical cord blood-derived MSC protect against ischemic acute kidney injury, a feature that is lost after inflammatory conditioning. J. Extracell. Vesicles.

[97] Collino F., Bruno S., Incarnato D., Dettori D., Neri F., Provero P., Pomatto M., Oliviero S., Tetta C., Quesenberry P.J. (2015). AKI Recovery Induced by Mesenchymal Stromal Cell-Derived Extracellular Vesicles Carrying MicroRNAs. J. Am. Soc. Nephrol..

[98] Collino F., Lopes J.A., Tapparo M., Tortelote G.G., Kasai-Brunswick T.H., Lopes G.M.C., Almeida D.B., Skovronova R., Wendt C.H.C., Miranda K.R. (2020). Extracellular Vesicles Derived from Induced Pluripotent Stem Cells Promote Renoprotection in Acute Kidney Injury Model. Cells.

[99] Eirin A., Zhu X.Y., Puranik A.S., Tang H., McGurren K.A., van Wijnen A.J., Lerman A., Lerman L.O. (2017). Mesenchymal stem cell-derived extracellular vesicles attenuate kidney inflammation. Kidney Int..

[100] Nassar W., El-Ansary M., Sabry D., Mostafa M.A., Fayad T., Kotb E., Temraz M., Saad A.N., Essa W., Adel H. (2016). Umbilical cord mesenchymal stem cells derived extracellular vesicles can safely ameliorate the progression of chronic kidney diseases. Biomater. Res..

[101] Zhu Z., Han C., Xian S., Zhuang F., Ding F., Zhang W., Liu Y. (2020). Placental Mesenchymal Stromal Cells (PMSCs) and PMSC-Derived Extracellular Vesicles (PMSC-EVs) Attenuated Renal Fibrosis in Rats with Unilateral Ureteral Obstruction (UUO) by Regulating CD4(+) T Cell Polarization. Stem Cells Int..

[102] Xia B., Crusius J., Meuwissen S., Pena A. (1998). Inflammatory bowel disease: Definition, epidemiology, etiologic aspects, and immunogenetic studies. World J. Gastroenterol..

[103] Valter M., Verstockt S., Finalet Ferreiro J.A., Cleynen I. (2020). Extracellular vesicles in inflammatory bowel disease: Small particles, big players. J. Crohns Colitis.

[104] Wang G., Yuan J., Cai X., Xu Z., Wang J., Ocansey D.K.W., Yan Y., Qian H., Zhang X., Xu W. (2020). HucMSC-exosomes carrying miR-326 inhibit neddylation to relieve inflammatory bowel disease in mice. Clin. Transl. Med..

[105] Cao L., Xu H., Wang G., Liu M., Tian D., Yuan Z. (2019). Extracellular vesicles derived from bone marrow mesenchymal stem cells attenuate dextran sodium sulfate-induced ulcerative colitis by promoting M2 macrophage polarization. Int. Immunopharmacol..

[106] Mao F., Wu Y., Tang X., Kang J., Zhang B., Yan Y., Qian H., Zhang X., Xu W. (2017). Exosomes Derived from Human Umbilical Cord Mesenchymal Stem Cells Relieve Inflammatory Bowel Disease in Mice. Biomed. Res. Int..

[107] Cheng M., Hu S., Wang Z., Pei Y., Fan R., Liu X., Wang L., Zhou J., Zheng S., Zhang T. (2016). Inhibition of neddylation regulates dendritic cell functions via Deptor accumulation driven mTOR inactivation. Oncotarget.

[108] Wu H., Fan H., Shou Z., Xu M., Chen Q., Ai C., Dong Y., Liu Y., Nan Z., Wang Y. (2019). Extracellular vesicles containing miR-146a attenuate experimental colitis by targeting TRAF6 and IRAK1. Int. Immunopharmacol..

[109] Koyama Y., Brenner D.A. (2017). Liver inflammation and fibrosis. J. Clin. Investig..

[110] Bruno S., Chiabotto G., Camussi G. (2020). Extracellular Vesicles: A Therapeutic Option for Liver Fibrosis. Int. J. Mol. Sci..

[111] Szabo G., Momen-Heravi F. (2017). Extracellular vesicles in liver disease and potential as biomarkers and therapeutic targets. Nat. Rev. Gastroenterol. Hepatol..

[112] Ohara M., Ohnishi S., Hosono H., Yamamoto K., Yuyama K., Nakamura H., Fu Q., Maehara O., Suda G., Sakamoto N. (2018). Extracellular Vesicles from Amnion-Derived Mesenchymal Stem Cells Ameliorate Hepatic Inflammation and Fibrosis in Rats. Stem Cells Int..

[113] Fiore E., Dominguez L.M., Bayo J., Malvicini M., Atorrasagasti C., Rodriguez M., Cantero M.J., Garcia M., Yannarelli G., Mazzolini G. (2020). Human umbilical cord perivascular cells-derived extracellular vesicles mediate the transfer of IGF-I to the liver and ameliorate hepatic fibrogenesis in mice. Gene Ther..

[114] Li X., Chen R., Kemper S., Brigstock D.R. (2019). Extracellular Vesicles From Hepatocytes Are Therapeutic for Toxin-Mediated Fibrosis and Gene Expression in the Liver. Front. Cell Dev. Biol..

[115] Bruno S., Pasquino C., Herrera Sanchez M.B., Tapparo M., Figliolini F., Grange C., Chiabotto G., Cedrino M., Deregibus M.C., Tetta C. (2020). HLSC-Derived Extracellular Vesicles Attenuate Liver Fibrosis and Inflammation in a Murine Model of Non-alcoholic Steatohepatitis. Mol. Ther..

[116] Chen L., Lu F.B., Chen D.Z., Wu J.L., Hu E.D., Xu L.M., Zheng M.H., Li H., Huang Y., Jin X.Y. (2018). BMSCs-derived miR-223-containing exosomes contribute to liver protection in experimental autoimmune hepatitis. Mol. Immunol..

[117] Jin Y., Wang J., Li H., Gao S., Shi R., Yang D., Wang X., Wang X., Zhu L., Wang X. (2018). Extracellular Vesicles Secreted by Human Adipose-derived Stem Cells (hASCs) Improve Survival Rate of Rats with Acute Liver Failure by Releasing lncRNA H19. EBioMedicine.

[118] Kawata R., Oda S., Koya Y., Kajiyama H., Yokoi T. (2020). Macrophage-derived extracellular vesicles regulate concanavalin A-induced hepatitis by suppressing macrophage cytokine production. Toxicology.

[119] Mardpour S., Hassani S.N., Mardpour S., Sayahpour F., Vosough M., Ai J., Aghdami N., Hamidieh A.A., Baharvand H. (2018). Extracellular vesicles derived from human embryonic stem cell-MSCs ameliorate cirrhosis in thioacetamide-induced chronic liver injury. J. Cell. Physiol..

[120] Yao J., Zheng J., Cai J., Zeng K., Zhou C., Zhang J., Li S., Li H., Chen L., He L. (2019). Extracellular vesicles derived from human umbilical cord mesenchymal stem cells alleviate rat hepatic ischemia-reperfusion injury by suppressing oxidative stress and neutrophil inflammatory response. FASEB J..

[121] Berenbaum F. (2013). Osteoarthritis as an inflammatory disease (osteoarthritis is not osteoarthrosis!). Osteoarthr. Cartil..

[122] Li Z., Wang Y., Xiao K., Xiang S., Li Z., Weng X. (2018). Emerging Role of Exosomes in the Joint Diseases. Cell Physiol. Biochem..

[123] Pourakbari R., Khodadadi M., Aghebati-Maleki A., Aghebati-Maleki L., Yousefi M. (2019). The potential of exosomes in the therapy of the cartilage and bone complications; emphasis on osteoarthritis. Life Sci..

[124] Tofino-Vian M., Guillen M.I., Perez Del Caz M.D., Castejon M.A., Alcaraz M.J. (2017). Extracellular Vesicles from Adipose-Derived Mesenchymal Stem Cells Downregulate Senescence Features in Osteoarthritic Osteoblasts. Oxid. Med. Cell. Longev..

[125] Zhang S., Teo K.Y.W., Chuah S.J., Lai R.C., Lim S.K., Toh W.S. (2019). MSC exosomes alleviate temporomandibular joint osteoarthritis by attenuating inflammation and restoring matrix homeostasis. Biomaterials.

[126] Vonk L.A., van Dooremalen S.F.J., Liv N., Klumperman J., Coffer P.J., Saris D.B.F., Lorenowicz M.J. (2018). Mesenchymal stromal/stem cell-derived extracellular vesicles promote human cartilage regeneration in vitro. Theranostics.

[127] Woo C.H., Kim H.K., Jung G.Y., Jung Y.J., Lee K.S., Yun Y.E., Han J., Lee J., Kim W.S., Choi J.S. (2020). Small extracellular vesicles from human adipose-derived stem cells attenuate cartilage degeneration. J. Extracell. Vesicles.

[128] He L., He T., Xing J., Zhou Q., Fan L., Liu C., Chen Y., Wu D., Tian Z., Liu B. (2020). Bone marrow mesenchymal stem cell-derived exosomes protect cartilage damage and relieve knee osteoarthritis pain in a rat model of osteoarthritis. Stem Cell Res. Ther..

[129] Albanesi C., Pastore S. (2010). Pathobiology of chronic inflammatory skin diseases: Interplay between keratinocytes and immune cells as a target for anti-inflammatory drugs. Curr. Drug Metab..

[130] Cho B.S., Kim J.O., Ha D.H., Yi Y.W. (2018). Exosomes derived from human adipose tissue-derived mesenchymal stem cells alleviate atopic dermatitis. Stem Cell Res. Ther..

[131] Shin K.O., Ha D.H., Kim J.O., Crumrine D.A., Meyer J.M., Wakefield J.S., Lee Y., Kim B., Kim S., Kim H.K. (2020). Exosomes from Human Adipose Tissue-Derived Mesenchymal Stem Cells Promote Epidermal Barrier Repair by Inducing de Novo Synthesis of Ceramides in Atopic Dermatitis. Cells.

[132] Kim W., Lee E.J., Bae I.H., Myoung K., Kim S.T., Park P.J., Lee K.H., Pham A.V.Q., Ko J., Oh S.H. (2020). Lactobacillus plantarum-derived extracellular vesicles induce anti-inflammatory M2 macrophage polarization in vitro. J. Extracell. Vesicles.

[133] He X., Dong Z., Cao Y., Wang H., Liu S., Liao L., Jin Y., Yuan L., Li B. (2019). MSC-Derived Exosome Promotes M2 Polarization and Enhances Cutaneous Wound Healing. Stem Cells Int..

[134] Whitcup S.M., Nussenblatt R.B., Lightman S.L., Hollander D.A. (2013). Inflammation in retinal disease. Int. J. Inflam..

[135] Klingeborn M., Dismuke W.M., Bowes Rickman C., Stamer W.D. (2017). Roles of exosomes in the normal and diseased eye. Prog. Retin. Eye Res..

[136] Li N., Zhao L., Wei Y., Ea V.L., Nian H., Wei R. (2019). Recent advances of exosomes in immune-mediated eye diseases. Stem Cell Res. Ther..

[137] Mathew B., Ravindran S., Liu X., Torres L., Chennakesavalu M., Huang C.C., Feng L., Zelka R., Lopez J., Sharma M. (2019). Mesenchymal stem cell-derived extracellular vesicles and retinal ischemia-reperfusion. Biomaterials.

[138] Shojaati G., Khandaker I., Funderburgh M.L., Mann M.M., Basu R., Stolz D.B., Geary M.L., Dos Santos A., Deng S.X., Funderburgh J.L. (2019). Mesenchymal Stem Cells Reduce Corneal Fibrosis and Inflammation via Extracellular Vesicle-Mediated Delivery of miRNA. Stem Cells Transl. Med..

[139] Tao H., Chen X., Cao H., Zheng L., Li Q., Zhang K., Han Z., Han Z.C., Guo Z., Li Z. (2019). Mesenchymal Stem Cell-Derived Extracellular Vesicles for Corneal Wound Repair. Stem Cells Int..

[140] Yu C., Chen P., Xu J., Liu Y., Li H., Wang L., Di G. (2020). hADSCs derived extracellular vesicles inhibit NLRP3inflammasome activation and dry eye. Sci. Rep..

[141] Zheng G., Huang R., Qiu G., Ge M., Wang J., Shu Q., Xu J. (2018). Mesenchymal stromal cell-derived extracellular vesicles: Regenerative and immunomodulatory effects and potential applications in sepsis. Cell Tissue Res..

[142] Chang C.L., Sung P.H., Chen K.H., Shao P.L., Yang C.C., Cheng B.C., Lin K.C., Chen C.H., Chai H.T., Chang H.W. (2018). Adipose-derived mesenchymal stem cell-derived exosomes alleviate overwhelming systemic inflammatory reaction and organ damage and improve outcome in rat sepsis syndrome. Am. J. Transl. Res..

[143] Bai X., Li J., Li L., Liu M., Liu Y., Cao M., Tao K., Xie S., Hu D. (2020). Extracellular Vesicles From Adipose Tissue-Derived Stem Cells Affect Notch-miR148a-3p Axis to Regulate Polarization of Macrophages and Alleviate Sepsis in Mice. Front. Immunol..

[144] Choi H., Kim Y., Mirzaaghasi A., Heo J., Kim Y.N., Shin J.H., Kim S., Kim N.H., Cho E.S., In Yook J. (2020). Exosome-based delivery of super-repressor IkappaBalpha relieves sepsis-associated organ damage and mortality. Sci. Adv..

[145] Wang L., Wang F.S., Gershwin M.E. (2015). Human autoimmune diseases: A comprehensive update. J. Intern. Med..

[146] Turpin D., Truchetet M.E., Faustin B., Augusto J.F., Contin-Bordes C., Brisson A., Blanco P., Duffau P. (2016). Role of extracellular vesicles in autoimmune diseases. Autoimmun. Rev..

[147] Kahmini F.R., Shahgaldi S. (2020). Therapeutic potential of mesenchymal stem cell-derived extracellular vesicles as novel cell-free therapy for treatment of autoimmune disorders. Exp. Mol. Pathol..

[148] Sharma J., Hampton J.M., Valiente G.R., Wada T., Steigelman H., Young M.C., Spurbeck R.R., Blazek A.D., Bosh S., Jarjour W.N. (2017). Therapeutic Development of Mesenchymal Stem Cells or Their Extracellular Vesicles to Inhibit Autoimmune-Mediated Inflammatory Processes in Systemic Lupus Erythematosus. Front. Immunol..

[149] Wang L., Gu Z., Zhao X., Yang N., Wang F., Deng A., Zhao S., Luo L., Wei H., Guan L. (2016). Extracellular Vesicles Released from Human Umbilical Cord-Derived Mesenchymal Stromal Cells Prevent Life-Threatening Acute Graft-Versus-Host Disease in a Mouse Model of Allogeneic Hematopoietic Stem Cell Transplantation. Stem Cells Dev..

[150] Fujii S., Miura Y., Fujishiro A., Shindo T., Shimazu Y., Hirai H., Tahara H., Takaori-Kondo A., Ichinohe T., Maekawa T. (2018). Graft-Versus-Host Disease Amelioration by Human Bone Marrow Mesenchymal Stromal/Stem Cell-Derived Extracellular Vesicles Is Associated with Peripheral Preservation of Naive T Cell Populations. Stem Cells.

[151] Guo L., Lai P., Wang Y., Huang T., Chen X., Geng S., Huang X., Luo C., Wu S., Ling W. (2020). Extracellular vesicles derived from mesenchymal stem cells prevent skin fibrosis in the cGVHD mouse model by suppressing the activation of macrophages and B cells immune response. Int. Immunopharmacol..

[152] Favaro E., Carpanetto A., Caorsi C., Giovarelli M., Angelini C., Cavallo-Perin P., Tetta C., Camussi G., Zanone M.M. (2016). Human mesenchymal stem cells and derived extracellular vesicles induce regulatory dendritic cells in type 1 diabetic patients. Diabetologia.

[153] Nojehdehi S., Soudi S., Hesampour A., Rasouli S., Soleimani M., Hashemi S.M. (2018). Immunomodulatory effects of mesenchymal stem cell-derived exosomes on experimental type-1 autoimmune diabetes. J. Cell. Biochem..

[154] Guo Q., Wang Y., Xu D., Nossent J., Pavlos N.J., Xu J. (2018). Rheumatoid arthritis: Pathological mechanisms and modern pharmacologic therapies. Bone Res..

[155] Tavasolian F., Moghaddam A.S., Rohani F., Abdollahi E., Janzamin E., Momtazi-Borojeni A.A., Moallem S.A., Jamialahmadi T., Sahebkar A. (2020). Exosomes: Effectual players in rheumatoid arthritis. Autoimmun. Rev..

[156] Cosenza S., Toupet K., Maumus M., Luz-Crawford P., Blanc-Brude O., Jorgensen C., Noel D. (2018). Mesenchymal stem cells-derived exosomes are more immunosuppressive than microparticles in inflammatory arthritis. Theranostics.

[157] Zheng J., Zhu L., Iok In I., Chen Y., Jia N., Zhu W. (2020). Bone marrow-derived mesenchymal stem cells-secreted exosomal microRNA-192-5p delays inflammatory response in rheumatoid arthritis. Int. Immunopharmacol..

[158] Meng Q., Qiu B. (2020). Exosomal MicroRNA-320a Derived From Mesenchymal Stem Cells Regulates Rheumatoid Arthritis Fibroblast-Like Synoviocyte Activation by Suppressing CXCL9 Expression. Front. Physiol..

[159] Casella G., Colombo F., Finardi A., Descamps H., Ill-Raga G., Spinelli A., Podini P., Bastoni M., Martino G., Muzio L. (2018). Extracellular Vesicles Containing IL-4 Modulate Neuroinflammation in a Mouse Model of Multiple Sclerosis. Mol. Ther..

[160] Riazifar M., Mohammadi M.R., Pone E.J., Yeri A., Lasser C., Segaliny A.I., McIntyre L.L., Shelke G.V., Hutchins E., Hamamoto A. (2019). Stem Cell-Derived Exosomes as Nanotherapeutics for Autoimmune and Neurodegenerative Disorders. ACS Nano.

[161] Li Z., Liu F., He X., Yang X., Shan F., Feng J. (2019). Exosomes derived from mesenchymal stem cells attenuate inflammation and demyelination of the central nervous system in EAE rats by regulating the polarization of microglia. Int. Immunopharmacol..

[162] Kim S., Maeng J.Y., Hyun S.J., Sohn H.J., Kim S.Y., Hong C.H., Kim T.G. (2020). Extracellular vesicles from human umbilical cord blood plasma modulate interleukin-2 signaling of T cells to ameliorate experimental autoimmune encephalomyelitis. Theranostics.

[163] Shigemoto-Kuroda T., Oh J.Y., Kim D.K., Jeong H.J., Park S.Y., Lee H.J., Park J.W., Kim T.W., An S.Y., Prockop D.J. (2017). MSC-derived Extracellular Vesicles Attenuate Immune Responses in Two Autoimmune Murine Models: Type 1 Diabetes and Uveoretinitis. Stem Cell Rep..

[164] Wang N., Ma J., Ren Y., Xiang S., Jia R. (2019). Secreted klotho from exosomes alleviates inflammation and apoptosis in acute pancreatitis. Am. J. Transl. Res..

[165] Deeks S.G., Tracy R., Douek D.C. (2013). Systemic effects of inflammation on health during chronic HIV infection. Immunity.

[166] Madison M.N., Roller R.J., Okeoma C.M. (2014). Human semen contains exosomes with potent anti-HIV-1 activity. Retrovirology.

[167] Naslund T.I., Paquin-Proulx D., Paredes P.T., Vallhov H., Sandberg J.K., Gabrielsson S. (2014). Exosomes from breast milk inhibit HIV-1 infection of dendritic cells and subsequent viral transfer to CD^4+^ T cells. AIDS.

[168] de Carvalho J.V., de Castro R.O., da Silva E.Z., Silveira P.P., da Silva-Januario M.E., Arruda E., Jamur M.C., Oliver C., Aguiar R.S., daSilva L.L. (2014). Nef neutralizes the ability of exosomes from CD^4+^ T cells to act as decoys during HIV-1 infection. PLoS ONE.

[169] Chung H.Y., Kim D.H., Lee E.K., Chung K.W., Chung S., Lee B., Seo A.Y., Chung J.H., Jung Y.S., Im E. (2019). Redefining Chronic Inflammation in Aging and Age-Related Diseases: Proposal of the Senoinflammation Concept. Aging Dis..

[170] Tsukamoto H., Kouwaki T., Oshiumi H. (2020). Aging-Associated Extracellular Vesicles Contain Immune Regulatory microRNAs Alleviating Hyperinflammatory State and Immune Dysfunction in the Elderly. Iscience.

[171] Wang W., Wang L., Ruan L., Oh J., Dong X., Zhuge Q., Su D.M. (2018). Extracellular vesicles extracted from young donor serum attenuate inflammaging via partially rejuvenating aged T-cell immunotolerance. FASEB J..

[172] Ellulu M.S., Patimah I., Khaza’ai H., Rahmat A., Abed Y. (2017). Obesity and inflammation: The linking mechanism and the complications. Arch. Med. Sci..

[173] Zhao H., Shang Q., Pan Z., Bai Y., Li Z., Zhang H., Zhang Q., Guo C., Zhang L., Wang Q. (2018). Exosomes From Adipose-Derived Stem Cells Attenuate Adipose Inflammation and Obesity Through Polarizing M2 Macrophages and Beiging in White Adipose Tissue. Diabetes.

[174] Pan Y., Hui X., Hoo R.L.C., Ye D., Chan C.Y.C., Feng T., Wang Y., Lam K.S.L., Xu A. (2019). Adipocyte-secreted exosomal microRNA-34a inhibits M2 macrophage polarization to promote obesity-induced adipose inflammation. J. Clin. Investig..

[175] Wasik M., Nazimek K., Nowak B., Askenase P.W., Bryniarski K. (2019). Delayed-Type Hypersensitivity Underlying Casein Allergy Is Suppressed by Extracellular Vesicles Carrying miRNA-150. Nutrients.

[176] Conlan R.S., Pisano S., Oliveira M.I., Ferrari M., Mendes Pinto I. (2017). Exosomes as Reconfigurable Therapeutic Systems. Trends Mol. Med..

[177] Tang T.T., Wang B., Lv L.L., Liu B.C. (2020). Extracellular vesicle-based Nanotherapeutics: Emerging frontiers in anti-inflammatory therapy. Theranostics.

[178] Gandham S., Su X., Wood J., Nocera A.L., Alli S.C., Milane L., Zimmerman A., Amiji M., Ivanov A.R. (2020). Technologies and Standardization in Research on Extracellular Vesicles. Trends Biotechnol..

[179] Yang D., Zhang W., Zhang H., Zhang F., Chen L., Ma L., Larcher L.M., Chen S., Liu N., Zhao Q. (2020). Progress, opportunity, and perspective on exosome isolation—Efforts for efficient exosome-based theranostics. Theranostics.

[180] Jafari D., Shajari S., Jafari R., Mardi N., Gomari H., Ganji F., Forouzandeh Moghadam M., Samadikuchaksaraei A. (2020). Designer Exosomes: A New Platform for Biotechnology Therapeutics. BioDrugs.

[181] Chen Y.S., Lin E.Y., Chiou T.W., Harn H.J. (2020). Exosomes in clinical trial and their production in compliance with good manufacturing practice. Ci Ji Yi Xue Za Zhi.

